# Interplay Between the Circadian Clock and Sirtuins

**DOI:** 10.3390/ijms252111469

**Published:** 2024-10-25

**Authors:** Yan Zhuang, Yantong Zhang, Chao Liu, Yingbin Zhong

**Affiliations:** 1 School of Basic Medical Sciences, Suzhou Medical College of Soochow University, Suzhou 215123, China; 2 School of Life Sciences, Suzhou Medical College of Soochow University, Suzhou 215123, China; 3 MOE Key Laboratory of Geriatric Diseases and Immunology, Suzhou Medical College of Soochow University, Suzhou 215123, China

**Keywords:** circadian clock, circadian rhythm, clock gene, sirtuin, metabolism

## Abstract

The circadian clock is an autonomous timekeeping system evolved by organisms to adapt to external changes, regulating a variety of important physiological and behavioral processes. Recent studies have shown that the sirtuin family of histone deacetylases is involved in regulating the expression of clock genes and plays an important role in maintaining the normal rhythm of clock gene expression and behavior. Moreover, sirtuins are regulated directly or indirectly by the circadian clock system. The mutual regulation between the circadian clock and sirtuins is likely involved in a variety of signal transduction and metabolism processes. In this review, we discuss the molecular mechanisms and research progress on the intertwined relationship between the circadian clock and sirtuins, mainly in mammals, highlighting sirtuins as molecular links between metabolic control and circadian rhythms and offering our perspectives on future developments in the field.

## 1. Introduction

The rotation of the Earth has resulted in daily changes in environmental factors, such as light, temperature, and food. This is a challenge that all life on Earth must face. To adapt to these daily environmental changes and efficiently utilize energy to maintain homeostasis, most organisms on Earth, including bacteria, algae, fungi, plants, and animals, have evolved a circadian clock system to anticipate and respond effectively to daily changes [1,2,3,4,5,6]. The circadian clock is able to run even under constant environmental conditions with an approximately 24 h periodicity. It regulates various physiological and behavioral activities, such as sleep, eating, hormone secretion, immunity, and cellular metabolism [7,8,9]. The circadian clock is genetically controlled, and alteration or disruption of the circadian clock can change the rhythmic physiology and behavior of animals and have detrimental effects on human health [10,11,12].

Sirtuins are a class of nicotinamide adenine dinucleotide (NAD^+^)-dependent histone deacetylases (HDACs) that play important biological roles within cells [13]. Acetylation and deacetylation are post-translational protein modifications which influence a myriad of cellular and physiological processes, protein stability and subcellular localization, enzyme activity and protein–protein interactions [14,15]. By remodeling the chromatin structure or gene expression, it is well known that reversible protein acetylation and its modifying enzymes have been proved to be important parts of epigenetics [14,16]. Sirtuins require NAD^+^ as a co-substrate. Firstly, sirtuins cleave NAD^+^ to nicotinamide (NAM) and, subsequently, the removal of each acetyl group form NAD^+^ results in the consumption of one molecule of NAD^+^ and the formation of nicotinamide and O-acetyl-ADP-ribose (OAADPr) [17,18]. This family was first named for its representative member in yeast, silent information regulator 2 (*Sir2*), and has been widely found in a wide range of organisms, including mammals [19]. Sir2 homologous proteins found in various species are collectively referred to as the sirtuin family, and the mammalian sirtuin family includes seven members (SIRT1-7) that have different subcellular localizations and functions [20]. Sirtuins regulate the acetylation state of not only histone, but also non-histone proteins. These proteins play important roles in processes such as cell apoptosis, mitochondrial biogenesis, lipid metabolism, fatty acid oxidation, cellular stress, insulin secretion, and aging [21,22].

Recent studies have shown that the sirtuin family, which regulates various energy metabolism pathways, is related to the expression of clock genes [23,24,25]. Moreover, the circadian clock directly regulates the rhythmic transcription of *Sirt1* by controlling its gene expression at specific times of the day. It also indirectly influences the activity of sirtuins by regulating the transcription of Nicotinamide phosphoribosyltransferase (*Nampt*), which is involved in NAD^+^ biosynthesis [26]. These findings implicate certain interactions between the circadian clock and sirtuins.

## 2. The Circadian Clock System and Its Molecular Architecture

The circadian clock system in mammals generally consists of three parts: the input or entrainment pathways, the central pacemaker or oscillator, and the output pathways [27]. The input pathway is responsible for sensing changes in external light and dark, temperature, and other time cues (so-called zeitgebers) and converting them into neural signals that are transmitted to the central pacemaker or oscillator. The central pacemaker or oscillator generates the molecular oscillations of clock genes and their related proteins in response to transmitted signals. Finally, the output pathway regulates downstream physiological and behavioral activities through these oscillations. In mammals, the circadian timekeeping system is composed of a central pacemaker in the suprachiasmatic nucleus (SCN) of the hypothalamus and subsidiary oscillators in most peripheral tissues [28,29,30]. The main zeitgeber of the central pacemaker is the periodic change in light and dark, whereas the zeitgeber of peripheral tissues is subjected mostly to cyclic feeding behavior [1,2,31,32]. The pacemaker SCN entrains to the environmental light/dark cycle via photic input from the retinohypothalamic tract (RHT). In turn, the SCN both directly and indirectly maintains the synchrony of peripheral oscillators through various entrainment mechanisms [31,32,33,34]. Furthermore, the peripheral circadian clock can independently regulate the expression of genes associated with tissue-specific functions, allowing their rhythms to exhibit a certain degree of tissue specificity [35,36].

**Figure 1:**
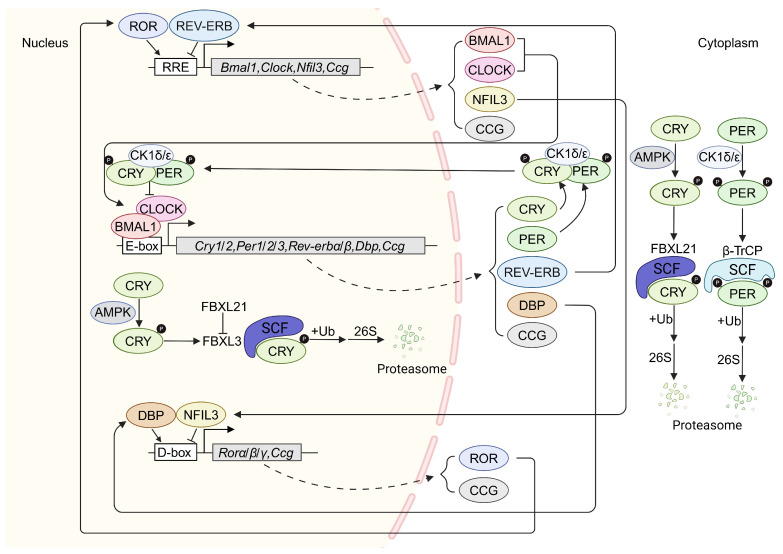
Molecular architecture of the core and interlocked feedback loops in mammals. At the core of these loops, BMAL1 and CLOCK form a heterodimeric transcriptional activator complex that binds to E-box motifs at promoters and enhancers to activate the transcription of the *Per1*, *Per2*, *Per3*, *Cry1*, and *Cry2* genes. PER and CRY form heterodimers and suppress their own transcription by inhibiting CLOCK-BMAL1 transcriptional activity. The second feedback loop is composed primarily of *Rev-erbα*, which serves as a direct target of the CLOCK-BMAL1 transcriptional activator complex. REV-ERBα provides negative feedback by inhibiting the transcription of *Bmal1* and competes with the retinoic acid-related orphan receptor (ROR) for binding to REV-ERB/ROR-binding elements (RREs) within the *Bmal1* promoter. In the last loop, NFIL3 and DBP inhibit and activate the expression of D-box genes, respectively, to regulate the rhythm of ROR nuclear receptors. All loops also control the expression of clock-controlled genes (*Ccgs*), which mediate circadian output. Selected factors that mediate post-translational modifications and degradation of specific clock proteins are shown. The arrows depict the synthesis, assembly, and/or localization of clock proteins; the blocked line denotes repression; BMAL1 is also known as ARNTL; CK1, casein kinase 1; AMPK, AMP-activated protein kinase; P, phosphorylation; E, E-box; RRE, REV-ERB/ROR response element; Ub, ubiquitylation.

The molecular architectures of the core circadian clock and peripheral tissue circadian clock are essentially the same. Both rely on interlocking transcription‒translation feedback loops (TTFLs) (Figure 1) [2,37,38]. In the core of these loops, the basic helix–loop–helix (bHLH)-Per-Arnt-Sim (PAS) transcription factors BMAL1 and CLOCK form heterodimers to promote the transcription of genes with an E-box (CACGTG) in their promoter and/or enhancer regions, including clock genes *Period* (*Per1*, *Per2*, *Per3*), *Cryptochrome* (*Cry1*, *Cry2*), *Rev-erbα/β*, *Rorα/β/γ*, and D-box binding protein gene (*Dbp*), thereby increasing the translation and accumulation of the corresponding proteins in the cytoplasm [39,40,41]. PER and CRY form a repressive complex with casein kinase 1δ/ε (CK1δ/ε), and the complex enters the nucleus and binds to CLOCK-BMAL1 heterodimers to form a tetra complex to inhibit the transcriptional activity of CLOCK-BMAL1, thereby repressing their own expression [42,43]. In the second interlocked loop, the REV-ERBα/β and RORα/β/γ proteins compete for binding to the REV-ERB/ROR-binding element (RRE) ([A/T]A[A/T]NT[A/G]GGTCA) in the promoter region, regulating the transcription of *Clock*, *Bmal1*, and *Nfil3* [44]. In the last interlocked loop, DBP activates while NFIL3 inhibits the expression of *Rorα/β/γ* through the D-box (TTA[T/C]GTAA), increasing the complexity of the entire negative feedback transcriptional regulatory mechanism [44,45,46,47]. These loops also control the expression of clock-controlled genes (*Ccgs*), which mediate circadian output [9,48,49,50] The maintenance of circadian rhythms by the evolutionary conserved circadian clock depends on the rhythmic expression of *Ccgs*, which depends significantly on cell and tissue types, the physiological and pathological state, as well as the criteria used for detecting rhythmicity [32,33].

In addition to transcriptional activators and repressors, the post-translational modifications and degradation of circadian clock proteins are key steps in the regulation of the circadian clock (Figure 1). The phosphorylation of PER proteins is mediated by casein kinase 1δ/ε (CK1δ/ε) [51,52], and these phosphorylation events regulate the sensitivity of PER to proteasomal degradation mediated by the E3 ligase β-TrCP (also known as F-box/WD repeat-containing protein 1A) [53,54]. Similarly, the phosphorylation of CRY1 and CRY2 induced by AMP-activated protein kinase (AMPK) also affects their stability [55]. Phosphorylated CRY1 and CRY2 become targets of the E3 ubiquitin ligases FBXL3 and FBXL21 and are ultimately degraded [56,57].

## 3. Sirtuins and Their Biological Functions

Sirtuins (SIRT1-7) all belong to the same category of HDACs, and the overall structure of the HDAC domain is similar in all isoforms. The structure of sirtuin proteins usually consists of two main domains: a large Rossmann fold domain and a small domain containing a zinc-binding module [58]. These two structural domains are highly conserved among all subtypes of the sirtuin family. The large Rossmann fold domain is related to NAD^+^ binding and catalytic acetyl group removal, whereas the small zinc-binding module provides support for protein stability and substrate binding [59]. These structural features constitute the key basis for sirtuin family proteins regulating various cellular processes [60]. The seven members of the mammalian sirtuin family have different subcellular localizations and regulate different cellular functions, with SIRT1 and SIRT2 being found both in the nucleus and the cytoplasm; SIRT6 and SIRT7 being nuclear proteins; and SIRT3, SIRT4 and SIRT5 being mitochondrial proteins, in a cell- and tissue-dependent context [61,62,63]. Studies have shown that SIRT1–3 have strong deacetylase activity, SIRT4–7 have weak or undetectable deacetylase activity, and SIRT4 mainly exhibits ADP-ribosyltransferase activity [64].

SIRT1 has the highest degree of homology with Sir2 and is the most comprehensively studied member of this family. It has classically been thought to be a nuclear protein, but studies have also shown that SIRT1 is localized to the cytoplasm as well [65]. SIRT1 interacts with histones and some non-histone substrates to regulate important physiological processes, such as glucose metabolism, fat metabolism, insulin secretion, angiogenesis, and cellular aging [66,67]. Additionally, SIRT1 plays a crucial role in mitigating oxidative stress and regulating redox signaling by enhancing antioxidant defenses and modulating the activity of key transcription factors involved in the oxidative stress response [68].

SIRT2 is localized mainly in the cytoplasm to deacetylate α-tubulin, and transiently migrates to the nucleus to deacetylate H4K16 during the G2/M transition [69,70]. Its functions involve regulating the cell cycle, neurodegeneration, tumor suppression, and inflammation [71,72,73]. Studies have shown that SIRT2 deacetylates FOXO1 and FOXO3 [74], which connect SIRT2 with DNA repair, apoptosis, metabolism, and aging [75]. SIRT2 also deacetylates PGC-1α, thereby modulating mitochondrial biogenesis [76].

SIRT3 is a key regulatory factor of mitochondrial function that is highly expressed in metabolically active tissues [77]. It is abundant in mitochondria and regulates energy production and the oxidative stress response [78,79]. SIRT3 directly interacts with glutamate dehydrogenase (GDH) and ornithine transcarbamylase (OCT) in mitochondria; regulates liver lipids, glycolysis, and the urea cycle; and protects the liver [80]. It indirectly regulates the accumulation of reactive oxygen species (ROS) by acetylating mitochondrial superoxide dismutase (SOD2) and isocitrate dehydrogenase 2 (IDH2), thereby inhibiting the transcriptional activity of hypoxia-inducible factor 1α (HIF-1α) and resisting oxidative stress [81]. It has been reported that the polymorphism of SIRT3 is related with longevity in humans [82,83]. But, studies from other groups do not support this finding [84,85].

SIRT4 is located in mitochondria, but it has weak deacetylase activity and relies mainly on its ADP-ribosyltransferase activity to regulate glutamine metabolism, thereby promoting the breakdown of branched-chain amino acids and fat formation and inhibiting insulin secretion and liver fatty acid oxidation [86,87]. Recently, SIRT4 was found to deacetylate lysine residues, controlling leucine metabolism and insulin secretion [88]. In addition, SIRT4 also regulates the DNA damage response and prevents the occurrence of tumors [89].

SIRT5 is also localized in the mitochondrial matrix, where it deacetylates, demalonylates, desuccinylates, and deglutarylates multiple proteins [90,91,92]. It is known for its ability to regulate mitochondrial fatty acid oxidation, the urea cycle, and cellular respiration [93,94]. SIRT5 has been shown to play roles in cellular metabolism, detoxification, the regulation of oxidative stress, energy production, and the mediation of the apoptosis pathway [95,96,97,98,99,100].

SIRT6 is a nuclear sirtuin. It has histone deacetylation and ADP ribosylation functions and plays an important role in chromatin regulation and gene expression [101,102,103,104]. SIRT6 is involved in DNA repair, antiinflammation, antioxidant defense, glucose metabolism, lipid metabolism, and cancer [105,106,107,108,109,110,111,112]. Studies on mouse models have revealed that SIRT6 is related to mouse lifespan [90,113,114].

SIRT7, the last member of the currently identified mammalian sirtuin family, is also located in the cell nucleus. It is specifically expressed in the nucleoli, where it deacetylates H3K18 and desuccinylates H3K122 to modulate chromatin remodeling, gene transcription, and DNA repair [115,116,117]. SIRT7 participates in cellular stress by inhibiting the activity of hypoxia-inducible factors [118], acts as an auxiliary factor for the inhibition of oncogene transcription [119], and plays a role in mammalian aging [90,120,121,122]. SIRT7 can also regulate some mitochondrial functions by deacetylating the GABP-beta 1 protein [123]. More biological functions of sirtuins have been well-discussed elsewhere [62,63,68,90,124].

## 4. Regulation of Sirtuins by the Circadian Clock

The circadian clock system, through the rhythmic expression of clock genes and clock-controlled genes, is widely involved in the regulation of various physiological processes, such as lipid metabolism, cholesterol synthesis, glucose metabolism and transport, and oxidative phosphorylation, and plays an important regulatory role in metabolism [125,126,127]. NAD^+^, a coenzyme of sirtuins, is a rhythmic metabolite regulated by the circadian clock that plays a crucial role in linking the circadian clock with sirtuins [128,129].

**Figure 2:**
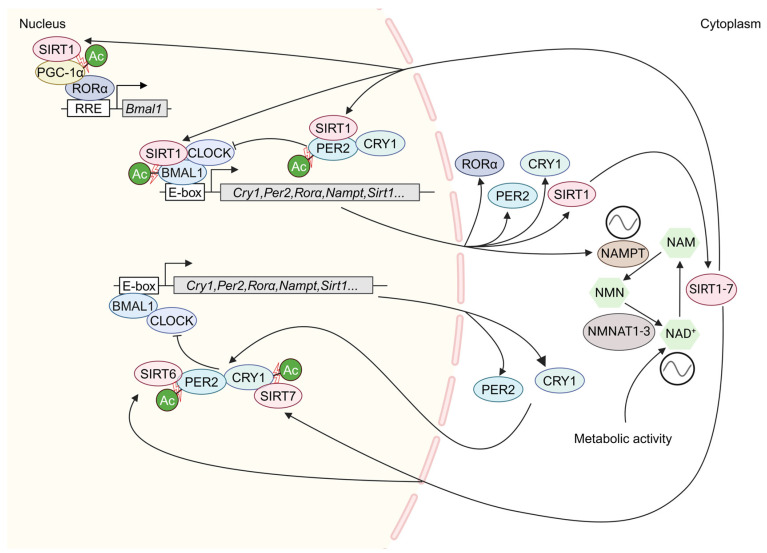
The mutual regulatory mechanisms between sirtuins and the circadian clock. NAD^+^ is a cellular energy sensor and sirtuins use NAD^+^ as a co-factor. The circadian clock regulates the activity of sirtuins through multiple mechanisms, including indirectly modulating sirtuin activity by controlling the rhythmic expression of *Nampt* to regulate NAD^+^ oscillation levels and directly influencing the transcription of the *Sirt1* or SIRT1 protein. SIRT1, in turn, interacts with CLOCK-BMAL1 to affect circadian rhythm amplitude and gene expression by deacetylating PER2, BMAL1, and histone H3 at circadian gene promoters. In contrast to SIRT1, SIRT6 interacts with CLOCK-BMAL1 and governs their chromatin recruitment to circadian gene promoters. SIRT6 can also affect the circadian clock by deacetylating PER2, whereas SIRT7 contributes to circadian clock regulation by deacetylating CRY1. NAM, nicotinamide; NMN, nicotinamide mononucleotide; NAMPT, nicotinamide phosphoribosyltransferase; NMNAT1-3, nicotinamide mononucleotide adenyltransferase; Ac, acetylation; dinusoidal lines represent rhythmic mRNAs; arrows depict the synthesis, assembly, and/or localization of clock proteins; the blocked line denotes repression.

The intracellular level of NAD^+^ is maintained by the tryptophan de novo synthesis pathway or the NAD^+^ salvage pathway controlled by the rate-limiting enzyme NAMPT [130,131]. Both the expression of *Nampt* RNA and the NAMPT protein display a diurnal pattern of oscillation [128,129]. Further study revealed that *Nampt* is a clock-controlled gene and that the circadian clock regulates the circadian expression of *Nampt* by binding the CLOCK-BMAL1 heterodimer to the promoter of *Nampt* (Figure 2) [128,129]. Therefore, the rhythmic expression of *Nampt* is the cause of circadian oscillation of NAD^+^ [128,129]. The specific pathway is as follows: the CLOCK-BMAL1 heterodimer binds to the E-box promoter element of the *Nampt* gene to promote *Nampt* expression; NAMPT catalyzes the rate-limiting step of the production of the intermediate nicotinamide mononucleotide (NMN) from NAM; and finally, this intermediate, NMN, is converted to NAD^+^ by NMNAT1-3 (Figure 2). Hence, the rhythmic activity of sirtuins is regulated by the oscillating levels of the intracellular metabolite NAD^+^, which emphasizes the importance of the circadian clock in the control of sirtuin activity and metabolism.

In addition to regulating *Nampt* transcription to affect sirtuin activity, the circadian clock can also directly regulate the transcription of *Sirt1* through the binding of the CLOCK-BMAL1 heterodimer to the E-box region of the *Sirt1* promoter [132]. The overexpression of the clock genes *Bmal1* and *Clock* in mouse liver cells can promote the accumulation of SIRT1, and interference with or mutation of *Clock* can decrease the expression levels of *Sirt1* mRNA and protein [132]. However, it has been reported that not *Sirt1* mRNA levels but rather SIRT1 protein levels accumulate in a circadian manner, with maximal and minimal levels reached at approximately zeitgeber time (ZT) 16 and ZT4 in the mouse liver, respectively [23]. These controversial findings require further investigation. Interestingly, SIRT1 is recruited to the *Nampt* promoter with CLOCK-BMAL1, thus contributing to the synthesis of its own coenzyme NAD^+^ [128]. In addition to SIRT1, whether other sirtuins are directly regulated by the circadian clock needs further investigation. Overall, regulation of sirtuin activity by the circadian clock highlights the close connection between circadian rhythms and metabolic control.

## 5. Regulation of the Circadian Clock by Sirtuins

Early research on cellular oxidation status demonstrated the impact of metabolism on the function of the circadian clock [133]. For example, the DNA binding activity of the CLOCK-BMAL1 heterodimer is affected by the intracellular redox state and the relative ratio of NAD(P)H/NAD(P)^+^, with high levels of NAD(P)H increasing the binding ability of CLOCK-BMAL1 to E-box elements [134].

Research has shown that the NAD^+^-dependent histone deacetylase SIRT1 directly regulates the acetylation status of clock components and affects the amplitude of circadian rhythms in cells and the liver (Figure 2 and Table 1) [135]. SIRT1 interacts with CLOCK and periodically regulates the acetylation of BMAL1 and histone H3, and the loss of *Sirt1* increases the amplitude of circadian rhythms [24]. These findings were confirmed by the pharmacological activation or inhibition of SIRT1 activity (Table 2) [24,128,129,136]. Moreover, inhibition of NAMPT activity by a specific chemical FK866 has effects on the circadian clock similar to those of the inhibition of SIRT1 (Table 2) [24,128,129]. These findings suggest that SIRT1 functions as a negative regulator of BMAL1 and circadian components. However, Asher et al. reported that SIRT1 deacetylates PER2 and promotes its degradation, and that the loss of *Sirt1* decreases the amplitude of circadian rhythms through PER2 acetylation [23]. Thus, SIRT1 functions as a positive regulator. Similar results were reported in neuronal cells, in which SIRT1 and PGC-1α bind cooperatively to the promoter of *Bmal1* to increase its expression amplitude [137]. These seemingly controversial results may be due to the different cell systems employed. In addition to its role in peripheral circadian control, SIRT1 also functions in the SCN. Brain-specific *Sirt1* knockout mice display reduced activity and prolonged periods of behavior rhythmicity, whereas brain-specific overexpression of *Sirt1* has the opposite effects [137]. Regarding the role of SIRT1 on BMAL1 and histone H3 acetylation, CLOCK itself possess acetyltransferase activity and can acetylate BMAL1 and histones, suggesting that SIRT1 and CLOCK may act together to maintain the balance of protein acetylation and contribute to epigenetic regulation [24,124,138]. Overall, these findings suggest that SIRT1 is physically associated with the CLOCK-BMAL1 complex [23,24], thereby participating in the regulation of the circadian clock feedback loop [128,129].

In contrast to SIRT1, SIRT6 interacts with CLOCK-BMAL1 and governs their chromatin recruitment to circadian gene promoters [139]. However, these two sirtuins interact independently with the clock machinery [139]. Thus, controlling circadian gene expression via SIRT6 and SIRT1 appears to define unique subdomains of oscillating CCGs that are involved in distinct biological functions [139]. Moreover, SIRT6 controls circadian chromatin recruitment of sterol regulatory element-binding protein-1 (SREBP-1), resulting in the cyclic regulation of genes implicated in fatty acid and cholesterol metabolism [139]. It has also been reported that SIRT6 interacts with and deacetylates PER2, preventing its proteasomal degradation, and the loss of *Sirt6* altered the oscillation of BMAL1 and PER2 mRNA and protein levels [140]. The opposite effects of SIRT1 and SIRT4 on PER2 may be due to the presence of multiple acetylation sites within the PER2 protein.

Finally, another sirtuin, SIRT7, has been reported to deacetylate CRY1 in mouse liver, thereby promoting its degradation to regulate the hepatic clock and glucose homeostasis [141].

**Table 1:** Effects of genetic manipulation of sirtuins on the circadian clock.

Gene	Manipulation	Cell/Tissue/Organism	Phenotype	References
*Sirt1*	SIRT1 overexpression	NIH3T3	Increased magnitude oscillation of *Bmal1*:, *Per2*:, and *Dbp*:*luciferase* reporter	[23]
			Decreased PER2 acetylation and increased PER2 degradation	[23]
		JEG3	Repressed CLOCK:BMAL1-driven *Per1*:*luciferase*	[136]
		HEK293	Suppressed CLOCK:BMAL1-driven *Per2:luciferase*	[129]
		Mouse primary hepatocytes	Suppressed expression and oscillation of *Per2*	[129]
	SIRT1 H363Y (enzyme activity dead mutation) overexpression	NIH3T3	Abolished oscillation of the *Bmal1*:*luciferase* reporter	[23]
	Brain-specific SIRT1 overexpression	Mouse	Increased behavior activity and shortened behavior period	[137]
			Restored ability to adapt to changes in light entrainment schedule in aged mice	[137]
		Mouse SCN	Upregulated expression of clock genes	[137]
	Knockdown	NIH3T3	Attenuated circadian oscillations of *Bmal1*:, *Per2*:, and *Dbp*:*luciferase* reporter	[23]
			Increased PER2 acetylation and protein level	[23]
		N2a	Reduced transcript and protein levels of clock genes	[137]
	Knockout	Mouse MEFs	Attenuated circadian oscillations of *Bmal1*-luciferase reporter, modest phase advance for the temporal expression of *Bmal1*:, *Per2*:, and *Dbp:luciferase*	[23]
			Reduced oscillation of *Bmal1*, *Clock*, *Per1*, *Cry1*, *Per2*, and *Rorγ* mRNA expression level	[23]
			Reduced oscillation of BMAL1 and CLOCK, and elevated PER2 and CRY1 protein level	[23]
			Increased PER2 acetylation and stability	[23]
			Increased transcription levels and broadened oscillation cycles of *Dpb* and *Per2*	[24]
			Increased and altered circadian histone H3 (Lys9/Lys14) acetylation levels	[24]
			Increased and altered circadian BMAL1 (Lys537) acetylation levels	[24]
	Liver-specific knockout	Mouse Liver	Increased and altered circadian BMAL1 (Lys537) acetylation levels	[24,136]
			Increased expression levels of CCGs *Dbp* and *Nampt* and enhanced oscillation of NAD^+^	[24,136]
	Brain-specific knockout	Mouse	Reduced behavior activity and elongated behavior period	[137]
			Reduced ability to adapt to changes in light entrainment schedule in young mice	[137]
			Downregulated expression of clock genes	[137]
*Sirt6*	Knockout	HEK293	Reduced PER2 protein level and stability and increased PER2 acetylation	[140]
		Mouse MEFs	Increased mRNA abundance of *Bmal1* and *Per2*	[140]
			Advanced phase of *Bmal1*, *Per2*, and *Cry1*	[140]
			Increased BMAL1 but decreased PER2 protein oscillation level	[140]
		Mouse liver	Reduced PER2 protein level	[140]
			Altered circadian transcriptome	[139]
			Enhanced association of BMAL1 to chromatin and increased circadian BMAL1 occupancy at *Dpb* and *Per1* promoter	[139]
			Increased Ac-H3K9 across all time points	[139]
			Highly enriched SREBP binding sites	[139]
			Altered circadian rhythmicity of genes and metabolites related to fatty acid metabolism	[139]
*Sirt7*	Knockout	Mouse liver	Advanced liver circadian phase Increased acetylation level of CRY1	[141]

**Table 2:** Effects of pharmacological manipulation of SIRT1 and NAMPT on the circadian clock.

Target	Manipulation	Medicine	Cell/Tissue	Phenotype	References
SIRT1	Inhibition	NAM	NIH3T3	Dampened circadian *Bmal1*:*luciferase* expression and lengthened circadian period	[23]
		Sirtinol	NIH3T3	Dampened circadian *Bmal1*:*luciferase* expression	[23]
		Splitomicin and NAM	Mouse MEFs	Increased transcription levels and broadened oscillation cycle of *dpb*	[24]
				Increased and altered circadian Ac-H3 (Lys9/Lys14) levels	[24]
		EX-527 or NAM	HEK293	Activated *Per2:luciferase* transcription	[129]
	Activation	SRT2183 and NAD^+^	Mouse MEFs	Decreased *Per2* transcription and amplitude	[136]
				Reduced Ac-H3 binding to *Per2* and *Dpb* promoter	[136]
		SRT1720	Mouse liver	Suppressed expression of *Per2*, *Dpb*, and *Nampt*	[136]
				Reduced recruitment of CLOCK and Ac-H3 on *Dbp* promoter	[136]
		SRTCD1023 and SRTCL1015	U2OS and NIH3T3	Reduced amplitude of both *Bmal1:* and *Per2*:*luciferase*	[136]
			Mouse MEFs	Repressed expression of *Per2* and *Cry1*	[136]
				Reduced CLOCK binding to *Per2* and *Dpb* promoter	[136]
		Resveratrol	HEK293	Inhibited *Per2:luciferase* transcription	[129]
NAMPT	Inhibition	FK866	Mouse MEFs	Advanced phase and increased amplitude of *Per2* and *Dbp*	[128]
				Increased and broadened perk of circadian Ac-BMAL1 (Lys537) levels	[128]
				Increased transcription *Per2*:*luciferase*	[129]
			Mouse primary hepatocytes	Damped *Per2* oscillation	[129]

## 6. Concluding Remarks

Although some interactions between the circadian clock and sirtuins have been established, there is still a limited understanding of the precise regulatory mechanisms involved. For example, in addition to SIRT1, the question remains whether other sirtuins are directly regulated by the circadian clock, and how. Are there any possible mechanisms by which sirtuins regulate the circadian clock? Interestingly, SIRT1, SIRT6, and SIRT7, which have been demonstrated to regulate the circadian clock by directly regulating clock gene expression or clock component acetylation, are all localized in the nucleus or cytoplasm [23,24,137,139,140,141]. However, the roles of the mitochondrial sirtuins SIRT3, SIRT4, and SIRT5 in the circadian clock have not been studied. The involvement of mitochondrial sirtuins in circadian regulation, as well as their regulatory mechanisms, remains to be further explored. Moreover, given the conserved function of sirtuins, their roles on the circadian cycle is limited in non-mammals. It has been reported that miR-92a targets *Drosophila Sirt2*, which is homologous to human *SIRT2* and *SIRT3*, modulating PDF neuronal excitability in a rhythmic manner [142].

Circadian control of metabolism is thought to be critical for organismal homeostasis [143,144]. Disruption of the circadian clock may lead to the occurrence of metabolic diseases, such as hyperglycemia, type 2 diabetes, and hypertension [46,145]. Metabolism is one of the regulatory targets of the circadian clock, which can also direct feedback to modulate clock function. Accumulating evidence reveals intriguing interactions between the circadian clock and cellular metabolism [146,147,148,149,150,151]. Sirtuins are particularly notable for their role in regulating cellular metabolism [63,152,153]. Consequently, sirtuins are considered potential therapeutic targets and are the focus of research related to metabolic diseases and aging [154,155,156]. The complex mutual regulatory process between sirtuins and the circadian clock suggests that there is an interlocked transcriptional‒enzymatic feedback loop that governs the molecular interplay between cellular metabolism and circadian rhythms [24,128,129]. More research is needed on the interaction between the circadian clock and sirtuins, as well as their potential therapeutic mechanisms in metabolic diseases. These studies will help elucidate the importance of the interplay between the circadian clock and sirtuins in maintaining metabolism homeostasis, providing new targets and strategies for the treatment of metabolic diseases.
